# A weekly 4‐methylpyrazole treatment attenuates the development of non‐obese metabolic dysfunction‐associated steatotic liver disease (MASLD) in male mice: Role of JNK

**DOI:** 10.1111/eci.14320

**Published:** 2024-09-29

**Authors:** Katharina Burger, Finn Jung, Raphaela Staltner, Katja Csarmann, Kerstin Schweiger, Annette Brandt, Anja Baumann, Julia Scholda, Florian Kopp, Ina Bergheim

**Affiliations:** ^1^ Department of Nutritional Sciences, Molecular Nutritional Science University of Vienna Vienna Austria; ^2^ Department of Pharmaceutical Sciences, Clinical Pharmacy Group University of Vienna Vienna Austria

**Keywords:** 4‐methylpyrazole, alcohol dehydrogenase inhibitor, c‐Jun N‐terminal kinase, endotoxin, inflammation

## Abstract

**Background:**

4‐methylpyrazole (4MP, fomepizole) is a competitive inhibitor of alcohol dehydrogenase (ADH) preventing the metabolism of ethylene glycol and methanol, respectively, into their toxic metabolites. 4MP seems also to possess a potential in the treatment of intoxication from other substance, for example, acetaminophen, and to modulate JNK‐dependent signalling. Here, we determined if a treatment with 4MP once weekly affects the development of diet‐induced non‐obese metabolic dysfunction‐associated steatotic liver disease (MASLD) in C57BL/6 mice.

**Methods:**

Male C57BL/6 mice (6–8 weeks old, *n* = 7‐8/group) were pair‐fed either a liquid control diet (C) or a liquid sucrose‐, fat‐ and cholesterol‐rich diet (SFC) for 8 weeks while being concomitantly treated with 4MP (50 mg/kg bw i.p.) or vehicle once a week. Liver damage, inflammatory markers and glucose tolerance were assessed. Moreover, in endotoxin‐challenged J774A.1 cells pretreated with 4MP, pro‐inflammatory markers were assessed.

**Results:**

The concomitant treatment of SFC‐fed mice with 4MP attenuated the increase in JNK phosphorylation and pro‐inflammatory markers like IFNγ, IL‐6 and 3‐nitrotyrosine protein adducts in liver tissue found in vehicle‐treated SFC‐fed mice, while not affecting impairments of glucose tolerance or the increase in portal endotoxin levels. Moreover, a pretreatment of endotoxin‐stimulated J774A.1 cells with 4MP significantly attenuated the increases in JNK phosphorylation and pro‐inflammatory mediators like IL‐6 and *Mcp1*.

**Conclusions:**

Taken together, our results suggest that a treatment with 4MP once weekly attenuates the activation of JNK and dampens the development of non‐obese MASLD in mice.

## Graphical abstract

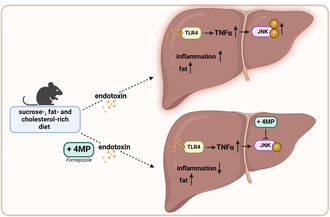

## 1 INTRODUCTION

By now, metabolic dysfunction‐associated steatotic liver disease (MASLD), formerly referred to as non‐alcoholic fatty liver disease (NAFLD), is thought to be the most prevalent liver disease worldwide.^1^ With numbers still increasing, results of epidemiological studies suggest that ~38% of the general population worldwide are affected by MASLD.^1^ Somewhat in contrast to the hypothesis that nutrition is only critical in the development through a general overnutrition, the number of non‐obese MASLD patients has been steadily increasing in recent years.^2^ Indeed, studies suggest that a diet rich in saturated fats, sugar and cholesterol may lead to the development of MASLD, even in the absence of marked overweight.^2^ Herein, alterations in the composition of intestinal microbiota and impairments of intestinal barrier function along with elevated bacterial endotoxin levels and a subsequent induction of Toll‐like receptor 4 (TLR4) in liver tissue have been shown to be critical.^3^ Also, studies suggest that elevated ethanol levels even in the absence of alcohol consumption stemming either from an enhanced synthesis by intestinal bacterial and/ or impaired alcohol dehydrogenase (ADH)‐dependent breakdown in the liver may also be related to the development of MASLD.^4^, ^5^ However, despite an ever increasing understanding of the changes of molecular signalling cascades associated with the development of MASLD and a first approved drug for treatment of patients with moderate to advanced fibrosis (stage 2 or 3),^6^ lifestyle interventions such as weight reduction and increased physical activity are still the main therapies for most MASLD patients.^7^

4‐methylpyrazole (4MP), also known as fomepizole, is an inhibitor of ADH.^8^ It is an approved medication (antidote) in the treatment of ethylene glycol and methanol intoxication, respectively, with only limited side effects.^9^ In more recent years, results of several studies have shown that 4MP may also be a drug in the treatment of acetaminophen intoxication (for overview, see^10^). Furthermore, it has been shown that 4MP may inhibit c‐Jun N‐terminal kinase (JNK), thereby reducing oxidative stress in mitochondria.^10^, ^11^ JNK has also been shown to be critical in the development of MASLD^12^ and seems to mediate the effects of TNFα with respect to neutrophil recruitment but also IL1ß and Il6 in liver tissue in the settings of non‐obese MASLD in mice.^13^

Starting from this background, the aim of the present study was to determine whether a treatment with 4MP once weekly alters the development of non‐obese MASLD in mice and if this is related to changes in JNK activation as well as to determine further associated molecular mechanisms.

## 2 METHODS

### 2.1 Animals and treatment

Male C57BL/6J mice (7–8 weeks old) were obtained from Janvier (Janvier SAS, Le‐Genest‐Saint‐Isle, France), respectively. Mice were kept in a specific pathogen‐free barrier facility accredited by the Association for Assessment and Accreditation of Laboratory Animal care. All procedures were approved and registered by the local Institutional for Animal Care and Use Committee (‘Bundesministerium für Wissenschaft, Forschung und Wirtschaft, Referat für Tierversuchswesen und Gentechnik’, Vienna, Austria; BMBWF‐66.006/0017‐V/3b/2019) and carried out under controlled conditions (12 h/12 h light/dark cycle, ~24°C, ~55% relative humidity) with mice having free access to tap water at all times. After adaptation to the facility, mice were randomly assigned to four groups (*n* = 6–8/group) and were pair‐fed either a liquid control diet (C, 69 E% carbohydrates, 12 E% fat, 19 E% protein) or a liquid sucrose‐, fat‐ and cholesterol‐rich diet (SFC, 55 E% carbohydrates, 30 E% fat derived from butterfat, 15 E% protein with 50% (wt./wt.) sucrose and 0.16% (wt./wt.) cholesterol; Ssniff, Soest, Germany) for 8 weeks as described in detail before.^13^ In brief, to achieve similar caloric intake within dietary groups, food intake per cage was determined daily and groups were adjusted accordingly in their caloric intake. Additionally, some of the mice received intraperitoneal (i.p.) injections of 50 mg/kg bw 4‐methylpyrazole (4MP (fomepizole), Sigma‐Aldrich, Steinheim, Germany) or vehicle (0.9% NaCl) once per week. Doses of 4MP were based on studies of others.^10^ In Week 7, a glucose tolerance test (GTT) was performed as described in detail before.^13^ At the end of the trial, mice were anaesthetised with a ketamine/xylazine mixture (i.p. injection, 100 mg ketamine/kg bw; 16 mg xylazine/kg bw) and killed by cervical dislocation. Blood was collected from portal vein and vena cava. Liver tissue samples were collected and fixed in neutral‐buffered formalin or immediately snap‐frozen and stored in a − 80°C freezer.

### 2.2 Cell culture

J774A.1 cells (DMSZ, Braunschweig, Germany) were cultured in Dulbecco's Modified Eagle Medium (PAN Biotech, Aidenbach, Germany) supplemented with 10% FBS and 1% penicillin/streptomycin (PAN Biotech, Aidenbach, Germany). In a first set of experiments, cells being 80% confluent were preincubated with 20 mM 4MP for 1 h and then treated with 50 ng/mL endotoxin (lipopolysaccharide (LPS), serotype O55:B5; Sigma‐Aldrich, Steinheim, Germany) for 18 h. In a second set of experiments, 80% confluent J774A.1 cells were pretreated with 2 mM 4MP and/ or 10 μM of the JNK inhibitor (SP600125, Thermo Fisher Scientific, MA, USA) for 1 h followed by a stimulation with 50 ng/mL endotoxin for 18 h. RNA was isolated as detailed below and supernatant was collected and stored at −80°C until further analysis. All cell culture experiments were repeated at least four to five times.

### 2.3 Statistical analysis

All data are presented as means ± standard error of the means (SEM). Statistical analysis was performed using PRISM (version 7.03, GraphPad Software, Inc.). Grubb's test was used to determine outliers before further statistical analysis. Homogeneity of variances was tested, and data were log‐transformed if data were not normally distributed or in case of inhomogeneity of variances before performing further statistical tests. A one‐ or two‐factorial analysis of variance (ANOVA) was used to determine the statistical differences between groups and a *p* < .05 was considered statistically significant. All further methods used are described in detail in online supplementary materials.

## 3 RESULTS

### 3.1 Effect of 4MP on markers of ethanol metabolism, liver damage and glucose tolerance

**FIGURE 1:**
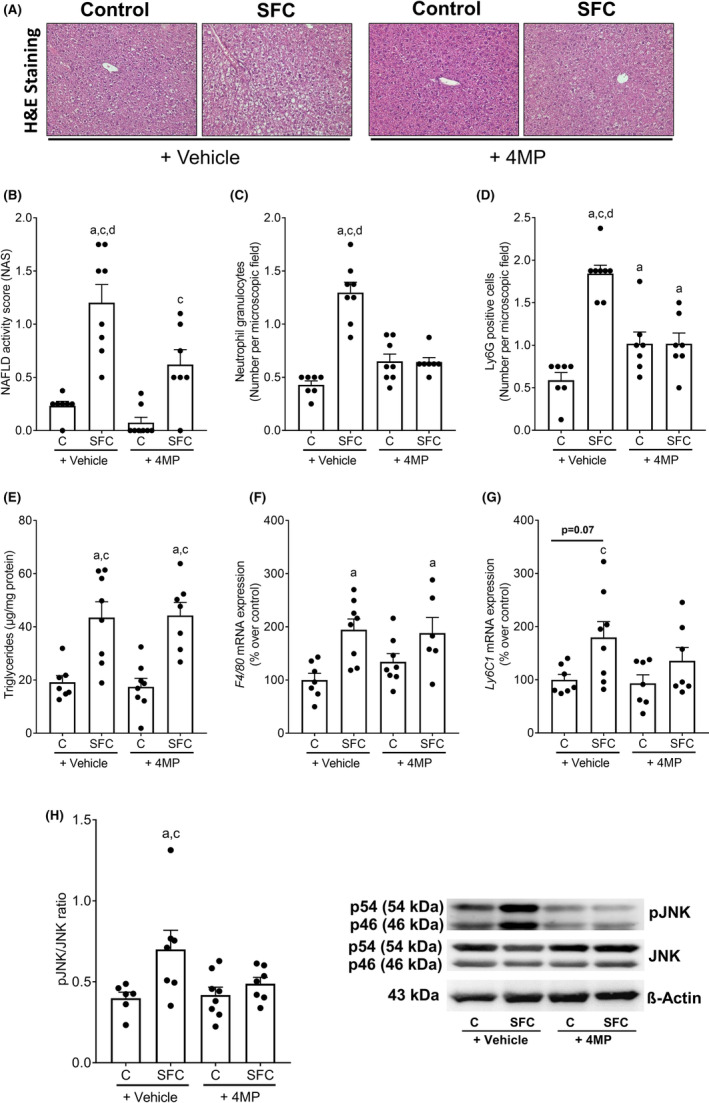
Effect of 4MP (50 mg/kg bw) on the development of MASLD and the phosphorylation of JNK in mice fed an SFC diet for 8 weeks. (A) Representative pictures of H&E staining (magnification 200×), (B) histological evaluation of liver sections using NAFLD activity score (NAS), (C) number of neutrophil granulocytes, (D) Ly6G‐positive cells per microscopic field, (E) triglycerides, (F) mRNA expression of *F4*/*80*, (G) *Ly6C1* as well as (H) phosphorylation of JNK protein in liver tissue and representative pictures of blots. Data are presented as means ± SEM, *n* = 7–8 except for (F) and (H) *n* = 6–8. ^a^ *p* <0.05 compared to C, ^c^ *p* <0.05 compared to C + 4MP, ^d^ *p* <0.05 compared to SFC + 4MP. C, control diet; Ly6c1, lymphocyte antigen 6 family member C1; Ly6G, lymphocyte antigen 6 complex locus G6D; SFC, sucrose‐, fat‐ and cholesterol‐rich diet; 4MP, 4‐methylpyrazole.

Absolute caloric intake was similar within SFC‐ and C‐fed groups but were significantly higher in SFC‐fed mouse groups compared to controls. Still, absolute body weight and weight gain were similar between all four feeding groups (Table 1). Neither ADH activity in cytosolic liver fraction nor ethanol levels in peripheral blood differed between C‐fed groups. Also, ADH activity was significantly lower in livers of both SFC‐fed groups to C‐fed mice but was similar between both SCF‐fed groups. In line with previous studies,^4^ ethanol levels in peripheral plasma were significantly higher in SCF‐fed mice compared to both C‐fed groups. In the 4MP treated SFC‐fed mice, ethanol concentration in peripheral blood was only significantly higher compared to C‐fed mice (Table 1). Cytochrome P450 (CYP) 2E1 activity in the microsomal fraction was similar in livers of vehicle‐treated C‐ and SFC‐fed mice but was lower in C‐ and SFC‐fed mice concomitantly treated with 4MP (C vs. C + 4MP: *p* = .055; SFC vs. C + 4MP and SFC + 4MP: *p* < .05) (Table 1). Despite not developing any signs of overweight, vehicle‐treated SFC‐fed mice developed clear signs of hepatic steatosis and beginning inflammation (Figure 1A and Table 1). Specifically, NAFLD activity score (NAS) and number of neutrophils in liver tissue were significantly higher in SFC‐fed mice than in controls. In SFC‐fed mice treated with 4MP signs of inflammation e.g. the number of inflammatory foci and of neutrophils were significantly lower than in vehicle‐treated SFC‐fed mice (Figure 1B,C). Also, numbers of lymphocyte antigen 6 complex locus G6D (Ly6G)‐positive cells were significantly higher in livers of SFC‐fed mice compared to all other groups. In livers of C‐ and SFC‐fed mice concomitantly treated with 4MP, numbers of Ly6G‐positive cells were higher than in vehicle‐treated controls (*p* < .05 for both) but did not differ between groups (Figure 1D).

**TABLE 1:** Effect of 4MP (50 mg/kg bw) on caloric intake, body weight, liver weight, markers of ethanol metabolism and glucose tolerance as well as endotoxin levels in mice fed a SFC diet for 8 weeks.

	+ Vehicle		+ 4MP	
	Control	SFC	Control	SFC
Caloric intake (kcal/g/bw)	10.0 ± 0.1	11.1 ± 0.1^a^, ^c^	9.9 ± 0.1	10.9 ± 0.2^a^, ^c^
Body weight (g)	28.9 ± 0.5	29.6 ± 0.6	28.1 ± 0.9	29.1 ± 0.6
Absolute body weight gain (g)	4.5 ± 0.3	4.5 ± 0.6	4.3 ± 0.4	4.9 ± 0.4
Liver weight (g)	1.4 ± 0.03	1.6 ± 0.04^a^, ^c^	1.3 ± 0.07	1.5 ± 0.1^c^
Liver: body weight ratio (%)	4.9 ± 0.1	5.5 ± 0.1^a^, ^c^	4.6 ± 0.1	5.3 ± 0.1^c^
ADH activity (mU/mL per 1000 cells) (% over control)	100.0 ± 7.3	72.0 ± 5.3^a^	83.7 ± 4.3	65.6 ± 7.2^a^
Ethanol (mM) (% over control)	100.0 ± 8.2	150.0 ± 9.2^a^, ^c^	109.0 ± 10.9	140.3 ± 5.8^a^
CYP2E1 activity (nmol p‐nitrocatechol/mg microsomal protein)	21.4 ± 1.2	23.5 ± 2.3^c^, ^d^	15.3 ± 1.3	13.5 ± 1.6^a^
ALT (U/L)	19.1 ± 1.8	15.5 ± 1.5	15.0 ± 1.3	14.6 ± 2.3
Fasting blood glucose (mg/dL)	125 ± 9	148 ± 3	128 ± 5	154 ± 4^a^
Area under the curve (AUC)	28,188 ± 1926	34,750 ± 1544^a^	29,820 ± 1554	38,968 ± 542^a^, ^c^
Bacterial endotoxin (OD 655 nm)	0.194 ± 0.01	0.338 ± 0.02^a^, ^c^	0.208 ± 0.01	0.320 ± 0.03^a^, ^c^

*Note*: Data are shown as means ± SEM, *n* = 6–8.

Abbreviations: 4MP, 4‐methylpyrazole; ADH, alcohol dehydrogenase; ALT, alanine aminotransferase; C, control diet; CYP2E1, Cytochrome P450 2E1; SFC, sucrose‐, fat‐, and cholesterol‐rich diet.

^a^ *p* <0.05 compared to C.

^c^ *p* <0.05 compared to C + 4MP.

^d^ *p* <0.05 compared to SFC + 4MP.

Hepatic steatosis and triglyceride levels were similar between SFC‐fed groups regardless of additional treatments. However, NAS in the SFC‐fed group treated with 4MP was still significantly higher compared to 4MP‐treated controls. Also, *F4/80* mRNA expression was similarly higher in livers of both SFC‐fed groups when compared to controls (Figure 1E,F). *Lymphocyte antigen 6 family member C1* (*Ly6C1*) expression was only higher in livers of vehicle‐treated SFC‐fed mice compared to controls (SFC vs. C: *p* = .07, SFC vs. C + 4MP: *p* < .05), while similar differences were not found when comparing 4MP‐treated SFC‐fed mice with controls (Figure 1G). Absolute liver weight and liver to body weight ratio were also significantly higher in both SFC‐fed mouse groups when compared to their respective controls. Alanine aminotransferase (ALT) activities in plasma were similar between all groups (Table 1). Furthermore, fasting glucose levels were by trend and significantly higher in SFC‐fed groups when compared to controls (C vs. SFC: *p* = .050, C vs. SFC + 4MP: *p* < .05) but were not different between SFC‐fed groups. Moreover, glucose tolerance as assessed by determining area under the curve (AUC) of blood glucose after a glucose challenge was also significantly higher in both SFC‐fed groups compared to control groups while not differing between both SFC‐fed groups (Table 1).

### 3.2 Effect of 4MP on phosphorylation of JNK in liver tissue

Phosphorylation of JNK was significantly higher in liver tissue of vehicle‐treated SFC‐fed mice compared to both control groups. In livers of SFC‐fed mice concomitantly treated with 4MP, phosphorylation of JNK was almost at the level of controls (Figure 1H).

### 3.3 Effect of 4MP on portal endotoxin levels and markers of inflammation as well as ER stress in liver tissue

**FIGURE 2:**
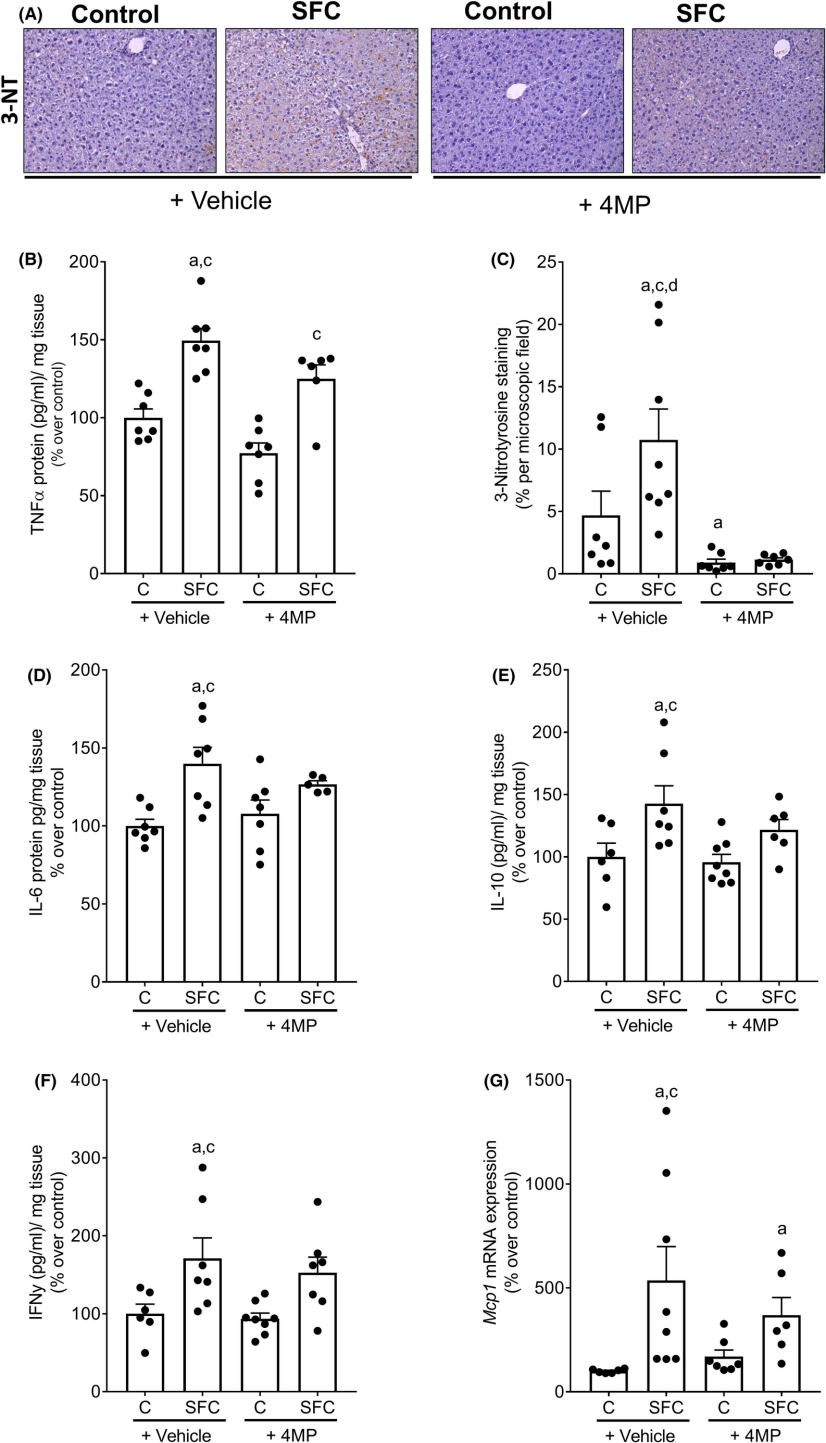
Effect of 4MP (50 mg/kg bw) on inflammatory markers in mice fed an SFC diet for 8 weeks. (A) Representative pictures of 3‐nitrotyrosine (3‐NT) protein staining (magnification 200×), (B) TNFα protein level and (C) densitometric analysis of 3‐NT protein staining in liver tissue. (D) IL‐6, (E) IL‐10 and (F) IFNγ protein levels as well as (G) mRNA expression of *Mcp1* in liver tissue. Data are presented as means ± SEM, *n* = 6–8 except for (B) *n* = 6–7, (D) *n* = 5–7, (E) *n* = 6–7. ^a^ *p* <0.05 compared to C, ^c^ *p* <0.05 compared to C + 4MP, ^d^ *p* <0.05 compared to SFC + 4MP. C, control diet; IFNγ, interferon gamma; IL, interleukin; Mcp1, monocyte chemoattractant protein‐1; SFC, sucrose‐, fat‐ and cholesterol‐rich diet; 3‐nitrotyrosine, 3‐NT; 4MP, 4‐methylpyrazole.

Bacterial endotoxin levels were significantly higher in both SFC‐fed groups regardless of additional treatments compared to control diet‐fed mice (Table 1). TNFα protein levels were also significantly higher in livers of both SFC‐fed mouse groups, regardless of additional treatments compared to their respective controls. In livers of controls treated with 4MP, protein concentration of TNFα was by trend lower than in vehicle‐treated controls (*p* = .053) (Figure 2B). Contrasting the findings for TNFα, concentration of 3‐nitrotyrosine (3‐NT) protein adducts was significantly higher in vehicle‐treated mice fed the SFC diet while being at the level of controls in 4MP‐treated SFC‐fed mice (Figure 2A,C). Also, protein levels of interleukin (IL)‐6, IL‐10 and interferon gamma (IFNγ) as well as mRNA expression of *intercellular adhesion molecule* (*Icam*) were only significantly higher in livers of vehicle‐treated SFC‐fed mice while no differences were found between 4MP‐treated SFC‐fed mice and all other groups when comparing these markers (Figure 2D–F and Table 2). *Monocyte chemoattractant protein 1* (*Mcp1*) mRNA expression was significantly higher in livers of vehicle‐treated SFC‐fed mice than in both control groups. In 4MP‐treated SFC‐fed mice, *Mcp1* mRNA expression was only significantly higher than in vehicle‐treated controls (Figure 2G). Expressions of the endoplasmic reticulum (ER) stress marker *glucose‐regulated protein 78* (*Grp78*) were significantly higher in livers of vehicle‐treated SFC‐fed mice when compared to their respective controls while differences alike were not found between groups treated with 4MP. *Spliced form of X‐box binding protein 1* (*Xbp1s*) mRNA expression was significantly higher in liver of both SFC‐fed groups when compared to vehicle controls whereas *C/EBP homologous protein* (*Chop*) mRNA expression was only significantly higher in liver of SFC‐fed mice concomitantly treated with 4MP when compared to controls (Table 2).

**TABLE 2:** Effect of 4MP (50 mg/kg bw) on expression of cell adhesion molecules and markers of hepatic ER stress in mice fed an SFC diet for 8 weeks.

	+ Vehicle		+ 4MP	
	Control	SFC	Control	SFC
*Icam* mRNA expression	100.0 ± 11.8	172.2 ± 23.0^a^, ^c^	106.5 ± 15.4	136.9 ± 9.3
*Grp78* mRNA expression	100.0 ± 16.8	298.1 ± 55.2^a^	309.2 ± 89.5	241.6 ± 52.7
*Xbp1s* mRNA expression	100.0 ± 26.4	344.2 ± 54.3^a^	236.6 ± 66.4	383.3 ± 123.7^a^
*Chop* mRNA expression	100.0 ± 15.9	199.9 ± 25.3	204.5 ± 34.7	233.0 ± 47.0^a^

*Note*: Data are shown as means ± SEM, *n* = 6–8.

Abbreviations: 4MP, 4‐methylpyrazole; C, control diet; Chop, C/EBP homologous protein; Grp78, glucose‐regulated protein 78; Icam, intercellular adhesion molecule; SFC, sucrose‐, fat‐ and cholesterol‐rich diet; Xbp1s, spliced form of X‐box binding protein 1.

^a^ *p* <0.05 compared to C.

^c^ *p* <0.05 compared to C + 4MP.

### 3.4 Effect of 4MP on endotoxin‐induced activation of J774A.1 cells

**FIGURE 3:**
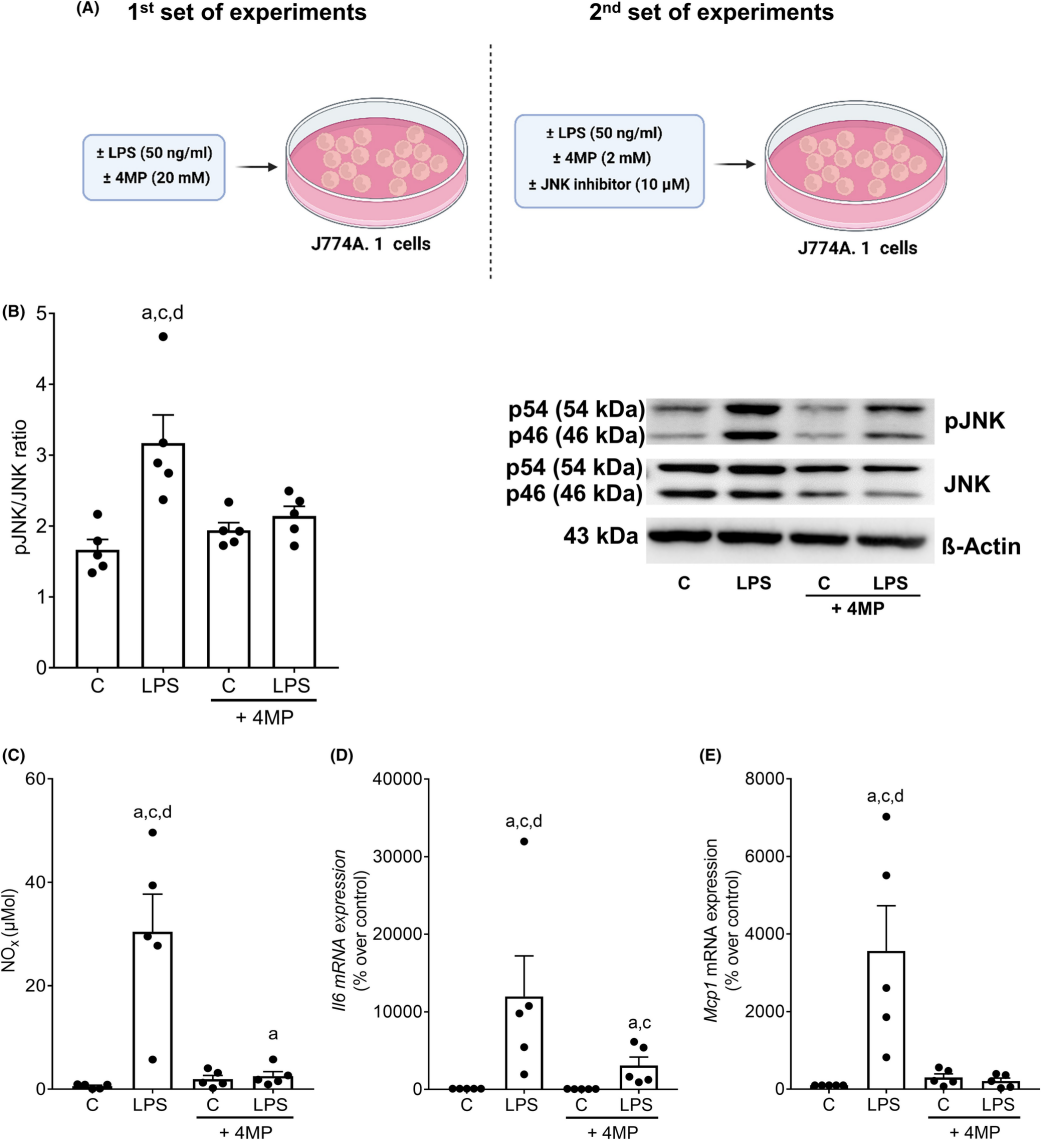
Effect of 4MP on the endotoxin induced activation and the phosphorylation of JNK in J774A. 1 cells. (A) Schematic drawing of the cell culture experiments, (B) phosphorylation of JNK protein in the cytosolic fraction and representative pictures of blots, (C) NO_x_ concentration in cell culture supernatant as well as mRNA expression of (D) *Il6* and (E) *Mcp1* in J774A.1 cells preincubated with 4MP (20 mM) for 1 h and stimulated with 50 ng/mL endotoxin for 18 h. Data are presented as means ± SEM, *n* = 5. ^a^ *p* <0.05 compared to C, ^c^ *p* <0.05 compared to C + 4MP, ^d^ *p* <0.05 compared to LPS + 4MP. C, untreated control cells; LPS, endotoxin‐stimulated cells; 4MP, 4‐methylpyrazole. Schematic drawing was created with BioRender.com.

**FIGURE 4:**
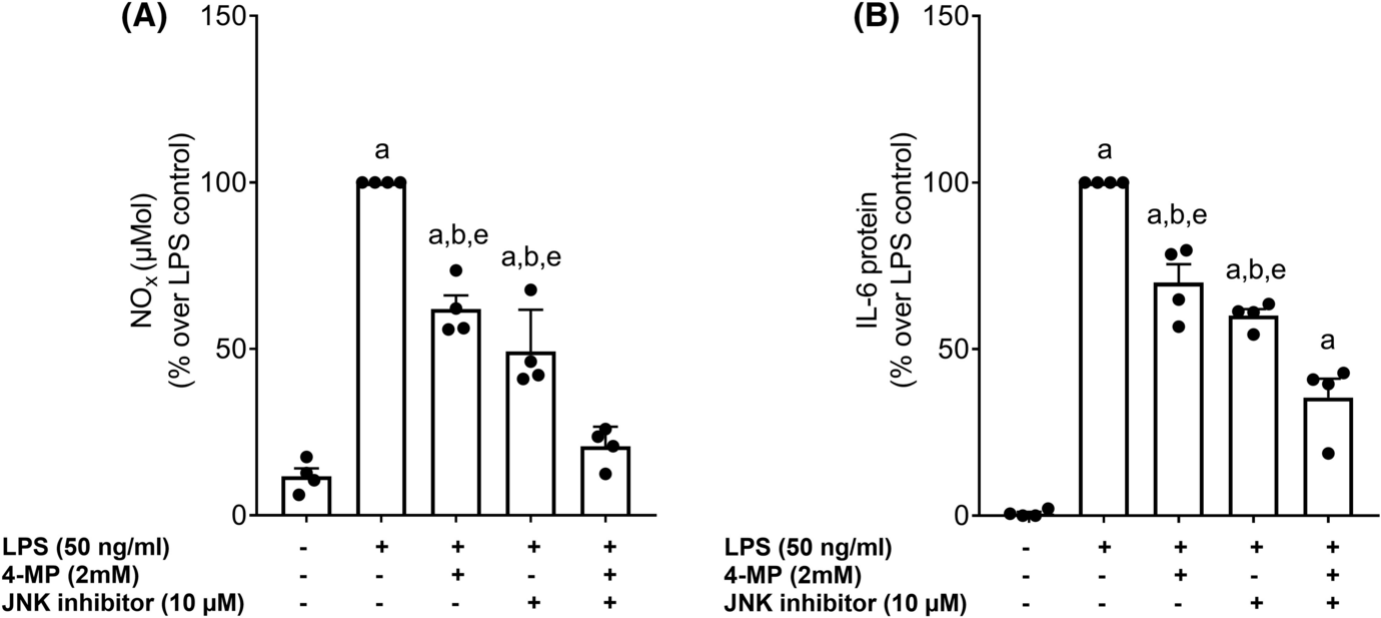
Effect of 4MP and a JNK inhibitor on the endotoxin‐induced activation in J774A.1 cells. (A) NO_x_ concentration and (B) IL‐6 protein levels in cell culture supernatant of J774A.1 cells preincubated with 4MP (2 mM) and a JNK inhibitor (SP600125, 10 μM) for 1 h followed by a stimulation with 50 ng/mL endotoxin for 18 h. Data are presented as means ± SEM; *n* = 4 ^a^ *p* <0.05 compared to C, ^b^ *p* <0.05 compared to endotoxin‐stimulated cells (LPS), ^e^ *p* <0.05 compared to endotoxin‐stimulated cells (LPS) preincubated with a JNK inhibitor and 4MP. C, untreated control cells; JNK, c‐Jun N‐terminal kinase; LPS, endotoxin‐stimulated cells; 4MP, 4‐methylpyrazole.

To further determine if 4MP also affects endotoxin‐dependent activation of immune cells and if this is related to JNK, J774A.1 cells, selected as in vitro model, were incubated with 20 mM 4MP before being challenged with endotoxin (for experimental set‐up, see Figure 3A). In line with the findings in vivo, in J774A.1 cells pretreated with 20 mM 4MP, the endotoxin‐dependent increase in pJNK was significantly blunted (Figure 3B). Furthermore, concentrations of nitric oxide (NO_x_) in supernatant and mRNA expressions of *Il6* and *Mcp1* were significantly higher in endotoxin‐treated cells compared to control cells and cells treated with 20 mM 4MP. While being significantly lower than in endotoxin‐treated cells, NO_x_ in supernatant and *Il6* mRNA expression in endotoxin‐treated cells pretreated with 4MP were still significantly higher than in naïve controls and, in the case of *Il6* mRNA expression, higher than in 4MP‐treated controls (Figure 3C–E). Moreover, in cells pretreated with the JNK inhibitor SP600125 in the presence of 4MP (2 mM), the endotoxin‐induced NO_x_ and IL‐6 protein release of J774.A1 cells into cell medium were attenuated in an almost additive manner (Figure 4A,B).

## 4 DISCUSSION

There is an increasing number of studies suggesting that specific dietary patterns, for example, those being rich in saturated fat, sugar and cholesterol, may even in the absence of overnutrition and weight gain, lead to the development of MASLD.^2^ In the present study, using a liquid pair‐feeding mouse model in which the control diet was based on standard chow and the MASLD‐inducing diet was sucrose‐, fat‐ and cholesterol‐rich, somewhat mimicking a ‘Western dietary pattern’ often associated in studies with the development of MASLD,^14^ mice developed early signs of MASH and insulin resistance in the absence of overweight or obesity. In line with previous studies^4^ the development of MASLD in the present study was associated with elevated ethanol levels in peripheral blood and lower ADH activity in liver tissue. Interestingly, neither was exaggerated in mice treated with the ADH inhibitor 4MP. Also, in controls, ethanol levels in peripheral blood were similar to vehicle‐treated controls as was ADH activity. Controls showed no signs of liver damage as determined by histology and ALT activity further suggesting that the treatment with 4MP once weekly had no adverse effects on the liver. It has been suggested before that 4MP when applied in humans in doses comparable to those used in the present study may decrease the rate of ethanol elimination by ~40% and that 4MP even at higher doses may only have limited adverse effects.^15^ However, if a regular long‐term ‘low’‐dose treatment with 4MP has adverse effect on either alcohol or retinoid metabolisms, the latter having been shown to be also mediated by ADH^16^ remains to be determined.

### 4.1 4MP treatment dampened the development of hepatic inflammation while not affecting fat accumulation and insulin resistance

In SFC‐fed mice concomitantly treated with 4MP, the development of inflammatory alterations was almost completely attenuated while both fat accumulation in the liver and insulin resistance were almost unaffected by the treatment with 4MP. The protective effects of 4MP were related to a protection from the activation of JNK as determined by assessing phosphorylation of JNK while bacterial endotoxin levels in portal plasma and TNFα protein levels as well as *F4*/*80* mRNA expression in liver were significantly higher in both SFC‐fed mouse groups. All other markers of inflammation in liver tissue (e.g. number of neutrophils and IL‐6, IL‐10, IFNγ, *Mcp1* mRNA expression and protein concentration, respectively, and 3‐nitrotyrosine protein adducts) were either at the level of controls or at least markedly lower when animals were treated with 4MP while being exposed to the SFC diet. Moreover, in line with studies of others,^17^ the treatment of mice with 4MP was related to a general inhibition of CYP2E1. CYP2E1 has been suggested to alter JNK activity through the formation of ROS.^18^, ^19^

However, results of our in vitro studies suggest that pretreating endotoxin‐stimulated J774.A1 cells with a JNK inhibitor in the presence of 4MP attenuated the increase of NO_x_ and IL‐6 in cell culture medium in an almost additive manner. Others also reported that 4MP may alter phosphorylation (=activation) of JNK through direct mechanisms.^20^ For instance, it has been shown before that 4MP prevents the activation of JNK in settings of acetaminophen‐induced liver damage in mice^10^, ^17^, ^20^ but also in t‐butylhydrogenperoxide‐induced oxidant stress as well as in a Gal/ET model of apoptosis and inflammatory liver injury.^20^ Moreover, results of these studies suggest that 4MP inhibited JNK phosphorylation and mitochondrial translocation.^17^, ^20^ Also, in these studies, the protective effects of 4MP were not related to a suppression of TNFα expression in liver tissue^20^ being in line with the findings in the present study and further supporting the hypothesis that 4MP may interact directly with JNK. Studies also suggest that the mitochondrial outer membrane protein SH3 homology‐associated BTK‐binding protein (SAB) plays a critical role in the activation of JNK by facilitating the release of ROS from mitochondria thereby maintaining JNK activation. In animal models, hepatic SAB expression is increased in mice fed a high caloric‐, fat‐ and fructose‐ rich diet promoting the release of ROS and leading to a sustained activation of the JNK pathway in a detrimental feedback loop.^21^ Whether the protective effects found in the present study were related to the effects of 4MP on CYP2E1 activity or a direct effect of 4MP on JNK and if SAB and/or ROS are critical herein remains to be determined.

In line with the present study showing limited effects of the 4MP treatment on the induction of hepatic TNFα expression and fat accumulation, results of studies employing in vitro and in vivo models have shown that TNFα can enhance fat accumulation in hepatocytes.^22^ Moreover, the number of F4/80‐positive cells being also similarly induced in livers of both SFC‐fed groups and being indicative of macrophages has also been shown to be related to TNFα signalling.^23^, ^24^ However, the lack of induction of *Ly6C1* in 4MP‐treated SFC‐fed mice suggests that the treatment with 4MP may have affected macrophage recruitment. Further studies are needed to determine if the lack of effectiveness of 4MP regarding hepatic fat accumulation found in the present study is related to its lack to attenuate the induction of TNFα or if other measures were critical in these alterations. Also, it remains to be determined how persistent the effects of the 4MP treatment are with respect to attenuating or diminishing inflammation in MASLD.

Moreover, in the present study, SFC‐fed mice treated with 4MP were not protected from the development of insulin resistance, suggesting that, in settings of diet‐induced non‐obese MASLD, other (TNFα‐dependent) signalling cascades bypassing JNK signalling may be critical in the development of insulin resistance. For instance, it has been shown that TNFα and other cytokines may also activate PTP1B which has been shown to block the IRS1/2‐dependet activation of AKT thereby subsequently leading to the development of insulin resistance.^25^ Moreover, it has been shown that TNFα may modulate insulin release in islet cells.^26^ However, if and how mechanisms alike were involved in the development of insulin resistance in the present study remains to be determined.

Concentrations of endotoxin in portal plasma discussed to be indicative of intestinal barrier function^3^ were almost similarly elevated in both SFC‐fed groups suggesting that 4MP did not affect intestinal barrier dysfunction. There are several studies suggesting that JNK may be critical in the development of intestinal barrier dysfunction of various aetiologies (e.g. chemical‐induced inflammatory bowel disease, sepsis‐related intestinal barrier dysfunction and osmotic stress‐induced related barrier disruption).^27^, ^28^, ^29^ However, it might be that intestinal barrier dysfunction related to the intake of specific nutrients, for example, sugar or fat, may not be related to an enhanced phosphorylation of JNK. This too needs to be explored in further studies.

While varying considerable within groups, markers of ER stress were also higher in both SFC‐fed groups. It could be that, in the present study, the enhanced release of TNFα in liver tissue was sufficient to still induce ER stress. Indeed, it has been shown before by others that TNFα derived from Kupffer cells is a critical mediator in ER stress and subsequently the development of overweight‐related MASLD.^30^ Taken together, results of our study suggest that, in mice, a regular treatment with 4MP attenuated inflammatory alterations related to the development of non‐obese MASLD. Further studies are needed to determine if these effects are persistent and also found in later stages of the disease.

## 5 CONCLUSION

In summary, results of the present study further bolster the hypothesis that 4MP is not only an effective ADH inhibitor and subsequently diminishes the metabolism of alcohols. Rather, in line with the findings of others,^17^, ^20^ results of our study suggest that a (low‐dose) treatment with 4MP may attenuate the activation of JNK and related signalling pathways and may thereby attenuate liver inflammation in settings of non‐obese MASLD. However, while results of our study suggest that 4MP did not exaggerate impairments of hepatic ethanol metabolism in mice with non‐obese MASLD, further studies are needed to determine the effect of 4MP on ethanol metabolism in settings of MASLD. Furthermore, taking the effects of 4MP on ethanol metabolisms in consideration, a use of 4MP in humans with MASLD will need careful consideration as MASLD patients may also drink alcohol. Still, results of the present study may open the field of application of 4MP also to other liver diseases related to a JNK activation and liver inflammation and add further weight to the hypothesis that an activation of JNK and dependent signalling cascades is critical in MASLD development.

## AUTHOR CONTRIBUTIONS

*Conceptualization*, Ina Bergheim; *data curation or formal analysis*, Katharina Burger, Finn Jung, Raphaela Staltner, Katja Csarmann, Kerstin Schweiger, Annette Brandt, Anja Baumann, Julia Scholda, Florian Kopp; *funding acquisition*, Ina Bergheim; *investigation*, Katharina Burger, Raphaela Staltner, Katja Csarmann, Kerstin Schweiger, Anja Baumann, Annette Brandt, Julia Scholda, Ina Bergheim; s*upervision*, Ina Bergheim; *visualization*, Katharina Burger, Ina Bergheim; *writing original draft preparation*, Katharina Burger and Ina Bergheim; *writing—review and editing*, Katharina Burger, Finn Jung, Raphaela Staltner, Katja Csarmann, Kerstin Schweiger, Annette Brandt, Anja Baumann, Florian Kopp and Ina Bergheim. All authors have read and agreed to the final version of the manuscript.

## FUNDING INFORMATION

This research received funding from the Austrian Science Fund (FWF, P 32164 (IB)).

## CONFLICT OF INTEREST STATEMENT

The authors disclose no conflicts of interest.

## Supporting information


**Data S1:** Supporting Information.

## Data Availability

Data can be made available upon reasonable request.
