# Supplementary material for: A weekly 4‐methylpyrazole treatment attenuates the development of non‐obese metabolic dysfunction‐associated steatotic liver disease (MASLD) in male mice: Role of JNK
Source: Eur J Clin Invest. 2024 Sep 29;55(1):e14320. doi: 10.1111/eci.14320 (PMC11628662; doi:10.1111/eci.14320)
Supplement: Supplementary file 1 — Data S1: Supporting Information. [file ECI-55-e14320-s001.pdf]

A weekly 4-methylpyrazole treatment attenuates the development of non-obese metabolic dysfunction-associated steatotic liver disease (MASLD) in male mice: Role of JNK

Katharina Burger[1], Finn Jung[1], Raphaela Staltner[1], Katja Csarmann[1], Kerstin Schweiger[1], Annette Brandt[1], Anja Baumann[1,] Julia Scholda[2], Florian Kopp[2], Ina Bergheim[1,*]

[1]Department of Nutritional Sciences, Molecular Nutritional Science, University of Vienna, Josef-Holaubek-Platz-2, 1090 Vienna, Austria

[2]Department of Pharmaceutical Sciences, Clinical Pharmacy Group, University of Vienna, Josef-Holaubek-Platz 2, 1090 Vienna, Austria

[*]Corresponding author: Ina Bergheim, Ph.D.
University of Vienna
Department of Nutritional Sciences
Molecular Nutritional Science
Josef-Holaubek-Platz 2
A-1090 Wien
Email address: ina.bergheim@univie.ac.at
Phone: +43-1-4277-54981
Fax: +43-1-4277-9549

Online supplementary materials. Additional methods used in the present study.

Evaluation of liver damage and inflammation

Paraffin-embedded liver sections (4 µm) were stained with hematoxylin and eosin (Sigma Aldrich Chemie GmbH, Steinheim, Germany) to evaluate liver histology using the non-alcoholic fatty liver disease (NAFLD) activity score (NAS) as detailed by Kleiner et. al. [1]. A commercially available naphthol AS-D chloroacetate esterase staining kit (Sigma-Aldrich Chemie GmbH, Steinheim, Germany) was used to stain neutrophil granulocytes as detailed previously [2]. Representative pictures were captured using a microscope with an integrated camera (LeicaDM4000 B LED, Leica, Wetzlar, Germany). Number of neutrophil granulocytes was determined per microscopic field in liver sections, for each tissue section a mean was determined from 8 fields (magnification 200 x). Alanine aminotransferase (ALT) activity in murine plasma was measured in a routine laboratory (Veterinary Medical University of Vienna, Vienna, Austria).

Immunostaining of 3-nitrotyrosine and Ly6G-positive cells

Using a polyclonal primary antibody (3-nitrotyrosine (3-NT); Santa Cruz Biotechnology, Dallas, TX, USA) as well as an monoclonal lymphocyte antigen 6 complex locus G6D (Ly6G) antibody (Abcam, Cambridge, UK), immunostainings of 3-NT protein adducts and Ly6G-positive cells were performed in liver tissue sections as detailed previously [3, 4]. To assess the staining intensity of 3-NT, defined as the percentage of the field area within the default color range, an analysis software incorporated in the microscope was used. Data were collected from eight pictures of each tissue section (magnification 200 x) using a microscope integrated camera (LeicaDM4000 B LED, Leica, Wetzlar, Germany). Ly6G-positive cells were counted in 8 microscopic fields and the mean was determined per liver section.

Western Blot

Liver tissue and cells were homogenized in lysis buffer (1 mol/L HEPES, 1 mol/L MgCl2, 2 mol/L KCl, and 1 mol/L dithiothreitol) containing protease and phosphatase inhibitors mix (Sigma-Aldrich Chemie GmbH) to obtain cytosolic protein lysates. Proteins lysates (30 µg/lane for cytosolic liver fraction; 7.5 µg/lane for cytosolic cell fraction from J774A.1 cells) were separated on 10% SDS-polyacrylamide gels and transferred to polyvinylidene difluoride membranes (Bio-Rad Laboratories, Hercules, CA, USA) as detailed before [5]. Membranes were further incubated with specific primary antibodies (phosphorylated c-Jun N-terminal kinase (JNK) and total JNK; Cell Signaling Technology, MA, USA; β-actin, Santa Cruz Biotechnology, Inc., TX, USA) and the respective secondary antibody (anti-rabbit IgG, HRP-linked or anti-mouse IgG, HRP-linked; Cell Signaling Technology, Massachusetts, USA). To detect the protein bands, the Super Signal Western Dura kit (Thermo Fisher Scientific, Waltham, MA, USA) was used, and densitometric analysis were performed using ChemiDoc XRS System (Bio-Rad Laboratories, Hercules, CA, USA) as detailed previously [6].

Enzyme-linked immunosorbent assays

Interleukin (IL)-6, IL-10, IFNγ and tumor necrosis factor alpha (TNFα) concentration in liver tissue or cell culture supernatant were measured using commercially available ELISA-Kits (DuoSet ELISA Kits, R&D Systems, Minneapolis, USA).

Griess assay

Nitric oxide (NO_X) levels in cell culture supernatant were measured using the Griess reagent assay according to the manufacturer (Promega GmbH, Madison, WI, USA).

RNA isolation, cDNA synthesis and real time PCR

Total RNA was extracted from liver tissue and cells using a commercially available kit (PeqGold Trifast, VWR International GmbH) and cDNA was synthesized (Reverse Transcription System, Promega GmbH, Madison, WI, USA) as detailed elsewhere [2].

To detect markers of endoplasmic reticulum (ER) stress, cDNA was synthesized using 400 ng of total RNA (Biozym cDNA Synthesis Kit,Biozym Scientific GmbH, Hessisch Oldendorf, Germany) and qRT-PCR was conducted using the Biozym Blue S'Green qPCR Kit (Biozym Scientific GmbH, Hessisch Oldendorf, Germany). Real-time polymerase chain reaction (PCR) was performed using primers listed in Table S1 to determine expression of the respective genes normalized to 18S as previously described [7].

Measurement of bacterial endotoxin

Concentration of bacterial endotoxin in portal plasma was measured using a commercially available reporter gene assay (InvivoGen, Toulouse, France, Cat.Number: mTLR4 = hkb-mtlr4), which assesses Toll-like receptor (TLR) 4 ligands, as detailed previously [8].

Ethanol levels in peripheral blood

Ethanol levels in murine plasma samples were measured using a commercially available kit (Ethanol FS, Diagnostic Systems GmbH, Holzheim, Germany).

ADH activity in liver

ADH activity was measured in cytosol isolated from whole liver tissue of mice as previously described [9].

CYP2E1 activity in liver

Cytochrome P450 enzyme (CYP) 2E1 activity was assessed in the microsomal fraction isolated from whole liver tissue as described by Cederbaum [10] and Chang et al [11]. In brief, microsomal fraction was isolated using ultracentrifugation as described previously [9]. Microsomal fraction was resuspended in Na_2HPO_4 (0.1M) buffered KCl (1.15 %). Activity of CYP2E1 was assessed in 200 µg of microsomal protein with p-nitrophenol as a substrate. Formation of p-nitrocatechol was determined at 535 nm.

Table S1: Primer sequences used for real-time PCR.

	Forward (5´- 3´)	*Reverse (5´-3´)*
18S	GTA ACC CGT TGA ACC CCA TT	CCA TCC AAT CGG TAG TAG CG
Chop	AGG AGA AGG AGC AGG AGA AC	AGA GAC AGA CAG GAG GTG ATG
F4/80	TGG CTG CCT CCC TGA CTT TC	CAA GAT CCC TGC CCT GCA CT
Grp78	CAC GTC CAA CCC CGA GAA	ATT CCA AGT GCG TCC GAT G
Icam	AGC TCG GAG GAT CAC AAA CG	CAG CCG AGG ACC ATA CAG CA
Il6	CCA CGC CTT CCC TAC TTC A	TGC AAG TGC ATC ATC GTT GTT C
Ly6c1	AGA AAG AGC TCA GGG ACT GC	AAA GAA AGG CAC TGA CGG GT
Mcp1	GTC CCT GTC ATG CTT CTG GG	GGC GTT AAC TGC ATC TGG CT
Xbp1s	GCT GAG TCC GCA GCA GGT	CAG GGT CCA ACT TGT CCA GAA T

Chop, C/EBP homologous protein; Grp78, glucose-regulated protein 78; Icam, intercellular adhesion molecule; Il, interleukin; Ly6c1, lymphocyte antigen 6 family member C1; Mcp1, monocyte chemoattractant protein 1; Xbp1s, spliced form of X-box binding protein 1

References

1. Kleiner DE, Brunt EM, Van Natta M, Behling C, Contos MJ, Cummings OW, Ferrell LD, Liu YC, Torbenson MS, Unalp-Arida A: **Design and validation of a histological scoring system for nonalcoholic fatty liver disease.** *Hepatology* 2005, **41:**1313-1321.

2. Spruss A, Kanuri G, Stahl C, Bischoff SC, Bergheim I: **Metformin protects against the development of fructose-induced steatosis in mice: role of the intestinal barrier function.** *Laboratory investigation* 2012, **92:**1020-1032.

3. Landmann M, Sellmann C, Engstler AJ, Ziegenhardt D, Jung F, Brombach C, Bergheim I: **Hops (Humulus lupulus) Content in Beer Modulates Effects of Beer on the Liver After Acute Ingestion in Female Mice.** *Alcohol Alcohol* 2017, **52:**48-55.

4. Sanchez V, Baumann A, Brandt A, Wodak MF, Staltner R, Bergheim I: **Oral Supplementation of Phosphatidylcholine Attenuates the Onset of a Diet-Induced Metabolic Dysfunction-Associated Steatohepatitis in Female C57BL/6J Mice.** *Cell Mol Gastroenterol Hepatol* 2024, **17:**785-800.

5. Burger K, Jung F, Baumann A, Brandt A, Staltner R, Sanchez V, Bergheim I: **TNFalpha is a key trigger of inflammation in diet-induced non-obese MASLD in mice.** *Redox Biol* 2023, **66:**102870.

6. Brandt A, Jin CJ, Nolte K, Sellmann C, Engstler AJ, Bergheim I: **Short-term intake of a fructose-, fat-and cholesterol-rich diet causes hepatic steatosis in mice: effect of antibiotic treatment.** *Nutrients* 2017, **9:**1013.

7. Sellmann C, Priebs J, Landmann M, Degen C, Engstler AJ, Jin CJ, Garttner S, Spruss A, Huber O, Bergheim I: **Diets rich in fructose, fat or fructose and fat alter intestinal barrier function and lead to the development of nonalcoholic fatty liver disease over time.** *J Nutr Biochem* 2015, **26:**1183-1192.

8. Jung F, Burger K, Staltner R, Brandt A, Mueller S, Bergheim I: **Markers of Intestinal Permeability Are Rapidly Improved by Alcohol Withdrawal in Patients with Alcohol-Related Liver Disease.** *Nutrients* 2021, **13**.

9. Burger K, Jung F, Staufer K, Ladurner R, Trauner M, Baumann A, Brandt A, Bergheim I: **MASLD is related to impaired alcohol dehydrogenase (ADH) activity and elevated blood ethanol levels: Role of TNFalpha and JNK.** *Redox Biol* 2024, **71:**103121.

10. Cederbaum AI: **Methodology to assay CYP2E1 mixed function oxidase catalytic activity and its induction.** *Redox Biol* 2014, **2:**1048-1054.

11. Chang TK, Crespi CL, Waxman DJ: **Spectrophotometric analysis of human CYP2E1-catalyzed p-nitrophenol hydroxylation.** *Methods Mol Biol* 2006, **320:**127-131.
